# Swimming Pool–Associated *Vittaforma*-Like Microsporidia Linked to Microsporidial Keratoconjunctivitis Outbreak, Taiwan

**DOI:** 10.3201/eid2511.181483

**Published:** 2019-11

**Authors:** Jung-Sheng Chen, Tsui-Kang Hsu, Bing-Mu Hsu, Shih-Chun Chao, Tung-Yi Huang, Dar-Der Ji, Pei-Yu Yang, I-Hsiu Huang

**Affiliations:** National Chung Cheng University, Minhsiung Township, Taiwan (J.-S. Chen, B.-M. Hsu, T.-Y. Huang);; Cheng Hsin General Hospital, Taipei, Taiwan (T.-K. Hsu);; Show Chwan Memorial Hospital, Changhua, Taiwan (S.-C. Chao, P.-Y. Yang);; National Chiao Tung University, Hsinchu, Taiwan (S.-C. Chao);; Central Taiwan University of Science and Technology, Taichung, Taiwan (S.-C. Chao);; National Yang-Ming University, Taipei (D.-D. Ji);; National Cheng Kung University, Tainan, Taiwan (I.-H. Huang)

**Keywords:** microsporidia, microsporidial keratoconjunctivitis, keratitis, swimming resort, Vittaforma-like microsporidia, *Vittaforma corneae*, transmission route, Taiwan, parasites

## Abstract

We analyzed 2 batches of environmental samples after a microsporidial keratoconjunctivitis outbreak in Taiwan. Results indicated a transmission route from a parking lot to a foot washing pool to a swimming pool and suggested that accumulation of mud in the foot washing pool during the rainy season might be a risk factor.

Microsporidia are obligate intracellular parasites that are generally found in aquatic environments (*1*,*2*). The main symptoms of microsporidiosis are keratoconjunctivitis, diarrhea, muscular infection, and acalculous cholecystitis, among which keratoconjunctivitis and diarrhea are the most common (*3*,*4*). As a result of improved diagnostic methods and increased awareness, microsporidia are now considered emergent pathogens worldwide (*4*,*5*). 

*Vittaforma corneae* has been considered a major risk factor for ocular microsporidiosis. In a previous study, we provided molecular evidence for the presence of *V. corneae* in hot springs in Taiwan (*6*). However, recent evidence indicates that ocular microsporidiosis might be underreported in keratoconjunctivitis (*5*,*7*). In our previous study, we hypothesized that *V. corneae* or *Vittaforma*-like microsporidia might spread from adjacent land environments (e.g., soil or mud) to aquatic environments (*2*). 

In June 2017, the New Taipei City Health Bureau (New Taipei City, Taiwan) was notified of a keratoconjunctivitis outbreak at a resort. The patients, healthy teenagers from a high school wrestling team, were found to contain DNA and spores from *V. corneae*, thus indicating that it was a microsporidial keratoconjunctivitis (MK) outbreak (*8*). Water contamination at the pool was suspected to be responsible for the outbreak. To identify the source of the pathogen, the transmission route, and the risk factors, water samples from various facilities at the swimming resort were collected for further evaluation. Moreover, because past studies have indicated that soil exposure is an important risk factor for MK, both soil and water samples were collected in a follow-up field survey. We describe results based on the 2 field surveys and provide information on the important risk factors for MK.

## The Study

This study was initially conducted because of a request of the New Taipei City Health Bureau in response to the MK outbreak (*8*). The swimming pools at the resort had been filled with tap water. Unfortunately, we received information about the MK outbreak 1 day after cleaning and disinfection of the swimming pools had taken place (*8*). Before water samples were collected, the resort was temporarily closed by the health authorities, and the facility was cleaned and disinfected as recommended by the health authorities (i.e., treatment with 5 ppm free available chlorine for 3 hours). The cleaning and disinfection procedures included draining of all water reservoirs, pools, and tubs, followed by surface scrubbing to remove any potential biofilms and disinfection with sodium hypochlorite. The pretreatment methods of samples, PCR conditions, phylogenetic analysis, and all protocols were performed as described in our previous studies (*2*,*6*).

We collected 19 water samples and 8 soil samples from the swimming resort and its surrounding environment (Appendix Figure 1). We sequenced all 17 amplicons of the 15 test-positive samples, analyzed them by using BLAST (http://blast.ncbi.nlm.nih.gov/Blast.cgi), and compared them with reference species from GenBank to determine the most closely related species. All the amplicons were homologous to microsporidia, with identity ranging from 89% to 99% (Table). Only (29.4%) amplicons showed a high degree of homology (>97% identity) to the reference strain.

**Table:** Comparison of BLAST results from environmental strains in a field study conducted after a microsporidial keratoconjunctivitis outbreak, Taiwan*

Strain	Sample source	Top BLAST hit (GenBank accession no.)	Maximum identity, study strain/reference (%)	Reference source
Xindian_1_Water	Standard swimming pool	*Vittaforma corneae* strain HotSpring-E1-o (KY245918)	470/473 (99)	Environmental
Xindian_3_Water	Standard swimming pool	*Vittaforma corneae* strain HotSpring-E1-o (KY245918)	467/473 (99)	Environmental
Xindian_10_Water	Foot wash pool	Microsporidium sp. BVOR4 (FJ756182)	438/472 (93)	Environmental
Xindian_14_Water	River	*Enterocytospora artemiae* isolate Ea_monica20 (JX915755)	477/483 (99)	*Artemia franciscana*
Xindian_15_Soil	Park near the resort	*Vittaforma corneae* strain HotSpring-C3-o (KY245925)	430/467 (92)	Environmental
Xindian_16_Water	Sink in the park	Uncultured microsporidia clone Chula_Myositis 1 (JN619406)	420/469 (90)	Clinical
Xindian_17_Soil	Park near the resort	*Nosema* sp. FCG-1468 (LC033883)	429/450 (95)	Honey bee
Xindian_18_Soil	Park near the resort	*Sporanauta perivermis* (KC172651)	432/485 (89)	Marine nematode
Xindian_20_Water	Wastewater from the resort	*Unikaryonidae* sp. JI-2011 (JF960137)	456/486 (94)	Curculionidae
Xindian_21_Water	Wastewater from the resort	Uncultured microsporidia clone Chula_Myositis 1 (JN619406)	425/474 (90)	Clinical
Xindian_22_Soil(U)	Soil in the wastewater flow	*Vittaforma corneae* strain HotSpring-F2-o (KY245925)	453/473 (96)	Environmental
Xindian_22_Soil(D)	Soil in the wastewater flow	*Vittaforma corneae* strain HotSpring-F2-o (KY245925)	450/472 (95)	Environmental
Xindian_23_Soil(U)	Park near the resort	*Vittaforma corneae* strain HotSpring-F3-o (KY245925)	458/472 (97)	Environmental
Xindian_23_Soil(D)	Park near the resort	*Vittaforma corneae* strain HotSpring-F2-o (KY245925)	448/474(95)	Environmental
Xindian_24_Soil	Parking lot near the resort	*Vittaforma corneae* strain LVPEI.BP235FR_11 (KP099409)	453/475 (95)	Clinical
Xindian_25_Water	Pavement rainwater	*Endoreticulatus* sp. Melnik (KU900486)	455/471 (97)	*Euproctis chrysorrhoea*
Xindian_26_Water	Foot wash pool	*Vittaforma corneae* strain LVPEI.BP235FR_11 (KP099409)	453/475 (95)	Clinical
Sw_MK_outbreak	Patient	*Vittaforma corneae* strain LVPEI.BP235FR_11 (KP099409)	472/472(100)	Clinical

*Top BLAST hit indicates the closest reference species that matched the environmental strains using BLAST search (http://blast.ncbi.nlm.nih.gov/Blast.cgi). Maximum identity represents the percentage identity between the environmental strains and the closest reference species. The fifth column shows the isolated source of the closest reference species.

In the initial survey (10 days after site disinfection), *V. corneae* was not detected. However, other *Vittaforma*-like microsporidia were identified in the standard swimming pool and foot washing pool. According to the Enforcement Rules for Swimming Pool Management in Taiwan, swimming pool water should be chlorinated (with a concentration of free available chlorine of ≈0.3–0.7 ppm) to prevent the spread of waterborne diseases. The presence of these other *Vittaforma*-like microsporidia indicates that some problems might have occurred during the disinfection process.

According to the New Taipei City Health Bureau, all pools, water source tanks, waterlines, and tubs in this facility were drained and scrubbed during the cleanup and disinfection process. The source of *V. corneae* infection remains debatable. Chlorine disinfection studies have shown that residual chlorine is capable of inactivating and reducing the number of microsporidians. However, microsporidian spores are relatively resistant to the typical concentrations of chlorine used for swimming pool disinfection (depending on the species, spores are inactivated after an exposure time of 10–120 min [i.e., inactivation of *Encephalitozoon intestinalis* spores does not reach 100% under 5 ppm of free available chlorine even after 120 minutes of treatment]) (*9*–*11*). Many clinical studies have indicated that soil or mud exposure, visits to hot springs, and outdoor activities, especially after rainfall, are all risk factors for ocular microsporidiosis (*12*,*13*). In addition, our previous study provided evidence for the presence of *V. corneae* in hot springs, thus indicating that pools in outdoor environments were associated with the presence of *V. corneae* (*6*). Therefore, we considered that the contaminating microsporidia in the swimming resort might have been brought to the resort from outside by human activities.

Our data show that many clade IV microsporidia were present in the soil and water samples from the resort site (Appendix Figure 2). Most of the microsporidia in clade IV are of terrestrial origin, according to small subunit rRNA gene phylogenetic analysis (*14*,*15*). Therefore, given that previous studies have shown that rainfall is an important risk factor for ocular microsporidiosis (*5*,*12*,*13*), we believe that water contamination might originate from soil environments after rainfall. Rainfall occurred near the sampling location during June 15–19 and again during July 1–4 (as recorded by the Taiwan Central Weather Bureau). We conducted a follow-up site survey after the July 1–4 rainfall and found *Vittaforma*-like microsporidians were found in pavement rainwater and the parking lot of the resort. Hence, we hypothesized that the swimming pool water was contaminated through soil or water brought in by human activity during the rainfall. *Vittaforma*-like microsporidians were found in the swimming pool and foot washing pool in the initial survey, which was conducted after a careful disinfection procedure, but microsporidia were not found in tap water or other pools. Therefore, these results suggest that the contamination was not from the waterlines or water sources, and the pools may have been contaminated from an outside source owing to human activities and poor facility configuration. In the follow-up survey, we found 100% identical amplicons in the parking lot and foot washing pool, suggesting a possible transmission pathway *Vittaforma*-like microsporidians from the outside environment to the swimming pool (Appendix Figure 1).

## Conclusions

Our study demonstrated the presence of *Vittaforma*-like microsporidia in a swimming resort and nearby environments in Taiwan. Human activities, rainy weather, and soil-rich or park environments might have been possible sources of microsporidia in the waters at the facility. The foot washing pool and shoe cabinet area are possible contamination areas and might facilitate transmission of microsporidia throughout the swimming resort. We suggest several precautions, including improving the frequency and efficacy of disinfection procedures at the facility, using a continuous water flow facility in foot washing pools, and paying attention to the disinfection and cleaning of the shoe cabinet area, especially during the rainy season. In addition, for swimming resorts that are located in a park, enhanced monitoring of the environment surrounding the swimming pool is warranted.

Appendix Additional information about swimming pool–associated *Vittaforma*-like microsporidia linked to microsporidial keratitis outbreak, Taiwan.
