# Supplementary material for: Swimming Pool–Associated Vittaforma-Like Microsporidia Linked to Microsporidial Keratoconjunctivitis Outbreak, Taiwan
Source: Emerg Infect Dis. 2019 Nov;25(11):2100–3. doi: 10.3201/eid2511.181483 (PMC6810191; doi:10.3201/eid2511.181483)
Supplement: Appendix — Additional information about swimming pool–associated Vittaforma-like microsporidia linked to microsporidial keratitis outbreak, Taiwan. [file 18-1483-Techapp-s1.pdf]

Article DOI: https://doi.org/10.3201/eid2511.181483

Swimming Pool–Associated *Vittaforma*-Like Microsporidia Linked to Microsporidial Keratitis Outbreak, Taiwan

Appendix

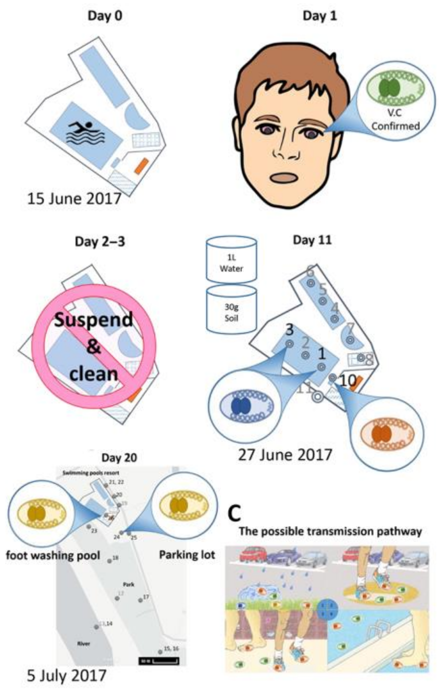

A

	Sample No.	Sampling Type
Initial survey	1	Standard swimming pool (Water)
	2	Standard swimming pool (Water)
	3	Standard swimming pool (Water)
	4	Spa pool
	5	Spa pool
	6	Spa pool
	7	Warm spa pool
	8	Bathing room
	9	Tap water source
	10	Foot wash pool (initial survey)
	11	Tap water source
Follow-up survey	12	Soil in the Park nearby the resort
	13	River (Soil)
	14	River (Water)
	15	Soil in the Park nearby the resort
	16	Sink in the park
	17	Soil in the Park nearby the resort
	18	Soil in the Park nearby the resort
	19	Wastewater from Rain flow
	20	Wastewater from the resort
	21	Wastewater from the resort
	22	Soil in the wastewater flow
	23	Soil in the Park nearby the resort
	24	Parking lot nearby the resort
	25	Pavement Rain water
	26	Foot wash pool(follow-up survey)

B

Sample type	First field survey (in resort)		Follow-up survey (around resort)	
	Total Samples	Number of detections	Total Samples	Number of detections
Standard swimming pool (Water)	3	2(66.7%)	0	--
Spa pool	3	0	0	--
Tap water source	2	0	0	--
Warm spa pool	1	0	0	--
Foot wash pool	1	1(100%)	1	1(100%)
Bathing room	1	0	0	--
River (Soil)	0	--	1	0
River (Water)	0	--	1	1(100%)
Sink in the park	0	--	1	1(100%)
Wastewater from the resort	0	--	2	2(100%)
Soil in the wastewater flow	0	--	1	1(100%)
Wastewater from Rain flow	0	--	1	0
Soil in the Park nearby the resort	0	--	5	4(80%)
Parking lot nearby the resort	0	--	1	1(100%)
Pavement Rain water	0	--	1	1(100%)
**Total**	**11**	**3(27.3%)**	**15**	**12(80%)**

Appendix Figure 1. Graphical abstract for this study. All patients had engaged in swimming activities at the resort on June 15 (Day 0). Some of the patients presented with keratoconjunctivitis symptom on June 16 (Day 1). The resort was temporally suspended, and full disinfection commenced (5 ppm free available chlorine for 3 hours) from June 17 to 18 (Days 2–3). The initial sample collection was performed on June 27 (Day 11). A follow-up sampling survey was conducted on July 5 (Day 20). Part (a) are the sampling locations of the swimming resort associated with microsporidial keratoconjunctivitis outbreak and its surrounding environment in Taiwan. Sampling location numbers 1 to 11 were in the swimming resort from the initial survey, and number 26 was in the swimming resort from the follow-up survey. The others were located at the park near the swimming resort from the follow-up survey. Part (b) is the occurrence of *Vittaforma*-like microsporidia in the water and soil samples from the two field surveys. Part (c) is the possible transmission pathway of microsporidians in the swimming resort. The illustration is presented from left to right and top to bottom. A person who goes swimming during or after a period of rainfall might carry microsporidian-contaminated soil or mud from the outside

environment of the park or other locations. Subsequently, when this person takes off his or her shoes and places them in the shoe cabinet, the cabinet might then be potentially contaminated. Then, the person with contaminated bare feet enters the foot washing pool, causing the foot washing pool to accumulate more contamination, especially as the foot washing pool in this resort had a low water flow rate. Finally, the standard swimming pool near the foot washing pool is contaminated by microsporidia, thus providing multiple sources for an ocular microsporidiosis outbreak.

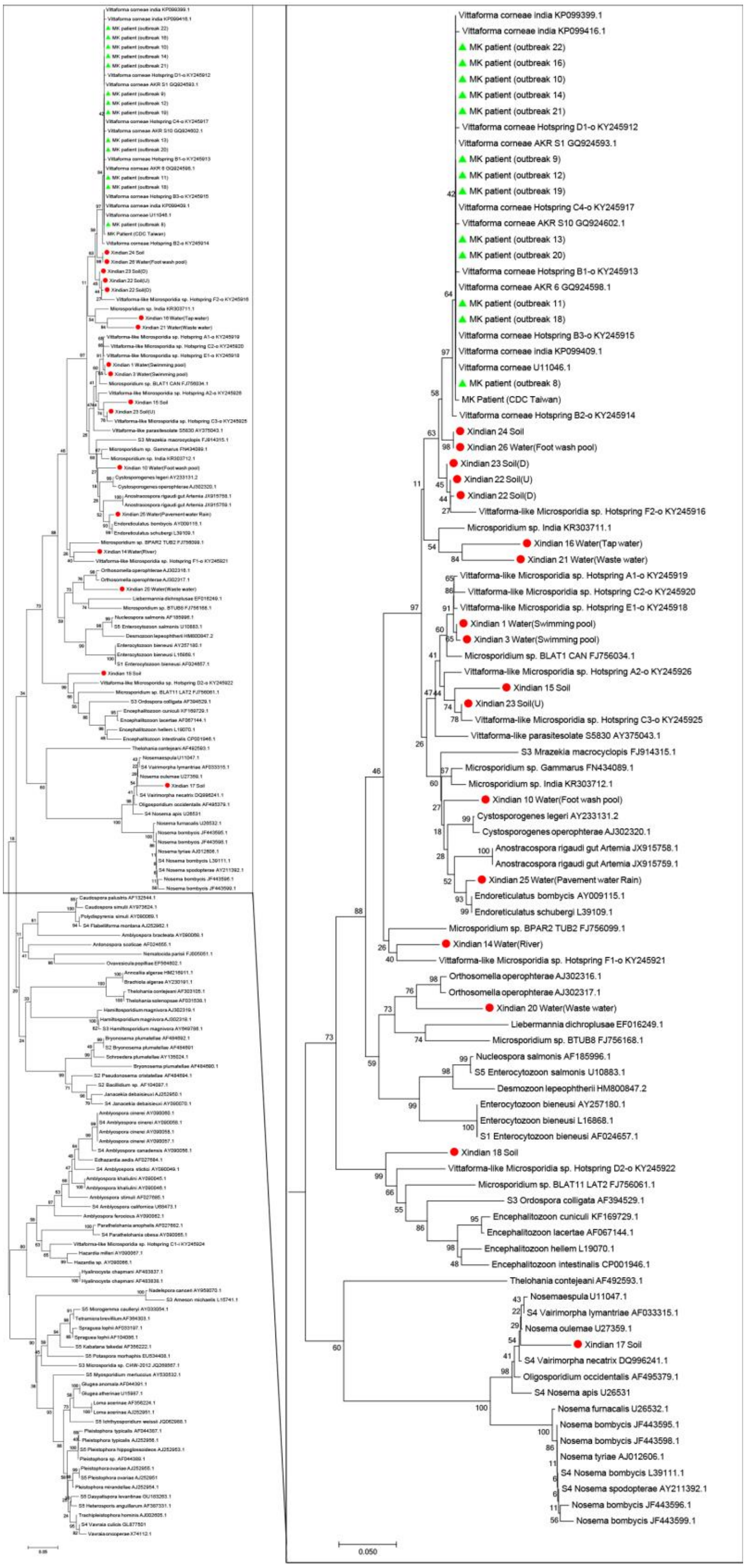

Appendix Figure 2. Phylogenetic analysis of the strains from this surveys. The phylogenetic tree was constructed based on the ≈472 bp SSU-rRNA gene sequences. Bootstrap values (%) involving 1000 pseudo-replicates for the major lineage are presented at each branch node. The major microsporidian clades are labeled by the black line on the left side of the figure. The right

part mostly consists of clade IV microsporidian sequences including our amplicons. The symbols -U, and -D indicate the amplicon position in the electrophoresis lane. The water or soil samples from this study are labeled by red circle and the microsporidial keratoconjunctivitis outbreak patients are labeled by green triangle. The reference strains were downloaded from GenBank with their accession numbers. Many reference strains cited in the literature are associated with five major clades of microsporidian sequences. The reference strains with symbol S before the species name are cited from Stentiford et al. (2013) (*1*) and are associated with aquatic animals' diseases according to GenBank. Number S1 represents fish-infecting taxa; S2 represents aquatic-arthropod–infecting taxa; S3 denotes non-arthropod invertebrate–, free-living protist–, and hyper-parasite–infecting taxa; S4 represents insect-infecting taxa; and S5 means human-infecting taxa.

Reference

1. Stentiford GD, Feist SW, Stone DM, Bateman KS, Dunn AM. Microsporidia: diverse, dynamic, and emergent pathogens in aquatic systems. Trends Parasitol. 2013;29:567–78. PubMed https://doi.org/10.1016/j.pt.2013.08.005
